# The “Dove Drawing” Appearance of the Ilium in Spondyloepiphyseal Dysplasia Congenita: A Proposed Radiographic Sign

**DOI:** 10.3390/diagnostics16193094

**Published:** 2026-09-24

**Authors:** Vladimir Kenis, Yuko Tsujioka, Gen Nishimura, Tatiana Markova, Evgeniy Melchenko, Daria Gorodilova

**Affiliations:** 1 H. Turner National Medical Research Center for Children’s Orthopedics and Trauma Surgery, Parkovaya 64–68, Pushkin, 196603 St. Petersburg, Russia; 2 North-Western State Medical University Named After I.I. Mechnikov, 191015 St. Petersburg, Russia; 3 Department of Radiology, Keio University School of Medicine, 35 Shinanomachi, Shinjuku 160-8583, Tokyo, Japan; 4 Department of Radiology, Musashino-Yowakai Hospital, 2-1-33 Midoricho, Musashino 180-0012, Tokyo, Japan; 5 Research Centre for Medical Genetics, Moskvorechye St., 1, 115522 Moscow, Russia

**Keywords:** *COL2A1*, spondyloepiphyseal dysplasia congenita, pelvic radiography, “dove drawing” sign

## Abstract

Recognizable radiographic signs aid in the differential diagnosis of skeletal dysplasias. Spondyloepiphyseal dysplasia congenita (SEDC), a COL2A1-related disorder, presents with characteristic but sometimes subtle pelvic changes in early childhood. To propose a novel pelvic radiographic sign observed in children with SEDC, we reviewed pelvic radiographs of a cohort of molecularly confirmed SEDC patients and noticed that the inferior contour of the ilium forms a continuous line resembling a flying dove, composed of the inner spur (resembling the dove’s beak), formed by the inferior contour of the iliac part of the sciatic notch, sharply angled to the most medial contour of the iliac part of triradiate cartilage, convex line (resembling the dove’s breast) composed of the lateral segment of the triradiate cartilage and medial segment of the acetabulum, and outer spur (resembling the junction between the body and wing) corresponding to the lateral part of the acetabulum and acetabular edge. The “dove drawing” sign may serve as an additional diagnostic clue for recognition of SEDC on pelvic radiographs, especially in children.

**Figure 1:**
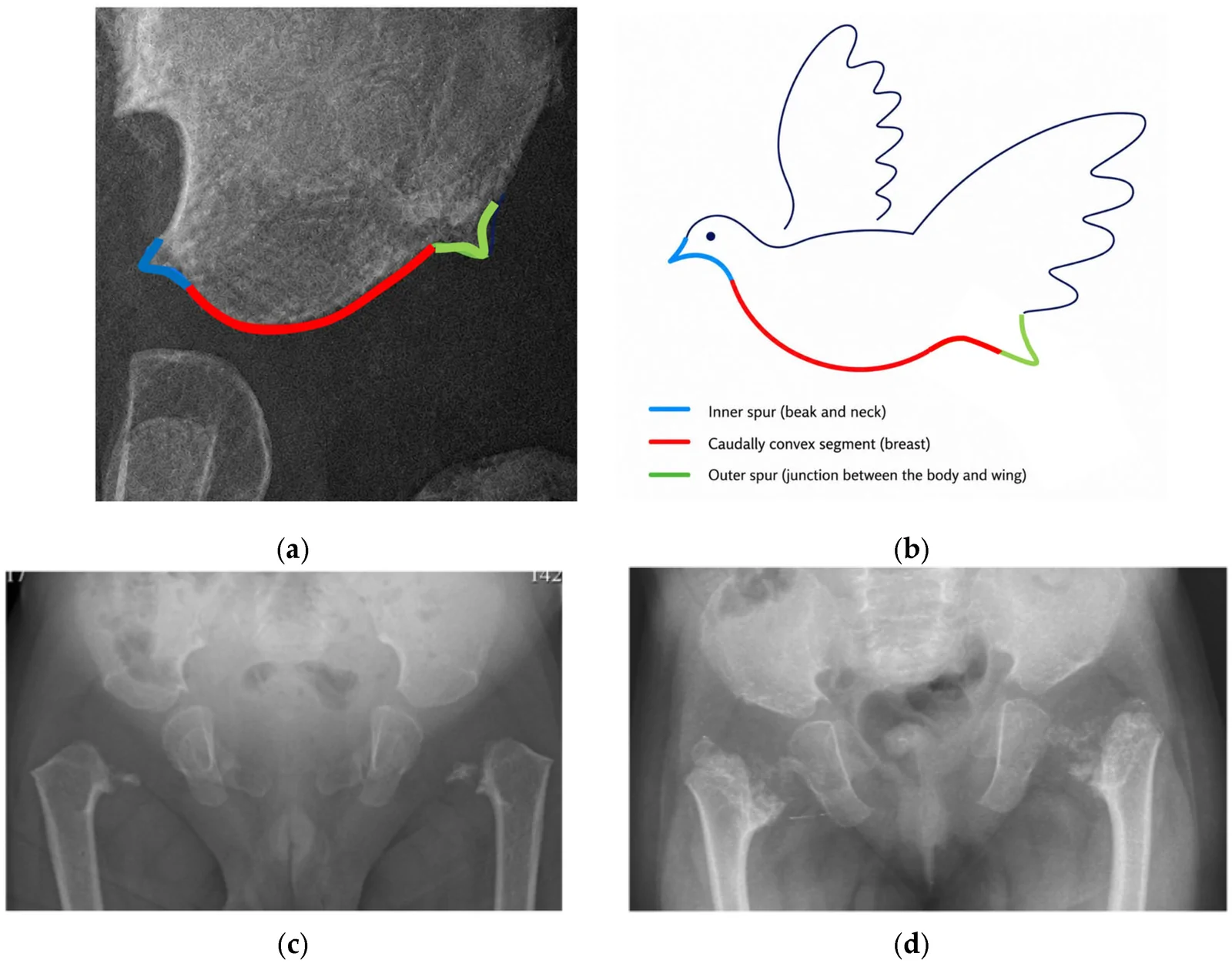
Despite the widespread use and increasing availability of molecular genetic testing, clinical and radiographic diagnosis of skeletal dysplasias remains essential. New radiographic phenomena continue to be described, particularly in rare skeletal diseases in children. The use of mnemonic aids and recognizable visual patterns greatly facilitates differential diagnosis [1]. Pelvic radiography is a valuable imaging modality for screening and characterizing skeletal dysplasias, called “window to skeletal dysplasias” [2]. Congenital spondyloepiphyseal dysplasia or spondyloepiphyseal dysplasia congenita (SEDC) is a part of the group of *COL2A1*-associated disorders, caused by pathogenic variants in the *COL2A1* gene, encoding the alpha-1 chain of type II collagen. In typical cases, it manifests with disproportionate short stature, abnormal epiphyses, and flattened vertebral bodies with manifestations presented at birth [3]. Key radiographic manifestations of SEDC in infancy include delayed ossification of the skeleton with absence of the ossification centers of the pubic bones and knee epiphyses, and defective ossification of the vertebral bodies of the upper cervical spine, as well as small, dorsally wedged vertebral bodies of the thoracolumbar spine, and delayed ossification of the sacrum. In childhood, radiographic findings include retarded ossification of the pelvis with horizontal acetabular roofs, absent or retarded ossification of the femoral head and neck, coxa vara (decreased angle between the partly ossified or anticipated femoral neck and the femoral shaft), varying degrees of epiphyseal and metaphyseal abnormalities of the long tubular bones, and delayed appearance of the carpal and tarsal ossification centers [4]. However, most of these signs can also be observed in other dysplasias and as normal variants; therefore, a combination of radiographic features is necessary to verify the diagnosis. In analyzing the radiographs of patients with COL2A1-associated skeletal dysplasias, we noted a characteristic feature of pelvic bone contours in children with SEDC. The radiographs were evaluated by consensus among all co-authors—orthopedic surgeons (V.K and E.M.), skeletal radiologists (G.N. and Y.T.), and clinical geneticists (T.M. and D.G.). Formal interobserver agreement was not calculated because of the descriptive nature of the article. The lines outlining the inferior contour of the ilium can be compared to the outline of a flying dove (**a**). We propose to refer to this as the “dove drawing” sign. The “dove drawing” is composed of the inner spur (resembling the dove’s beak), formed by the inferior contour of the iliac part of the sciatic notch, sharply angled to the most medial contour of the iliac part of triradiate cartilage (marked in blue). The drawing continues laterally with the convex line (resembling the dove’s breast) composed of the lateral segment of the triradiate cartilage and medial segment of the acetabulum (marked in red), and the outer spur (resembling the junction between the body and wing) corresponding to the lateral part of the acetabulum and acetabular edge (marked in green) (**b**). This continuous line is demonstrated on the anteroposterior radiographs of 1-year-old (**c**) and 3-year-old (**d**) children with SEDC. Radiographs in the present report are partly overlapped with the authors’ previous publication [5] with principally different anatomical accents. In the typically developing children, neither the inner nor outer spur is seen, and the acetabular roof is concave. The outer and inner spurs of the “dove drawing” may hypothetically correspond to the metaphyseal corner spurs of long bones that are commonly seen in type 2 collagenopathy [6], although the connection between abnormal COL2A1 and these radiographic features is not clearly understood. The convexity of the triradiate cartilage segment and acetabulum is similar to the convexity of the bone ends seen in type 2 collagenopathy, particularly Kniest dysplasia and Stickler dysplasia [7], which may reflect a broader COL2A1-related skeletal phenotype rather than being specific to SEDC. Pelvic radiography is one of the most informative methods for screening skeletal dysplasias. Striking and memorable semiotic signs are widely known in clinical practice (for example, the “trident sign” in achondroplasia, caused by the endochondral ossification features of the iliac bones [8], multiple or “absent” ossification of the pubic bones in SEDC or cleidocranial dysplasia respectively [5,9]). The “dove drawing” sign may serve as an additional diagnostic clue, facilitating recognition of SEDC during routine radiographic evaluation.

## Data Availability

The original contributions presented in this study are included in the article. Further inquiries can be directed to the corresponding author.
